# Architectural Heterogeneity in Tumors Caused by Differentiation Alters Intratumoral Drug Distribution and Affects Therapeutic Synergy of Antiangiogenic Organoselenium Compound

**DOI:** 10.1155/2010/396286

**Published:** 2010-04-27

**Authors:** Youcef M. Rustum, Károly Tóth, Mukund Seshadri, Arindam Sen, Farukh A. Durrani, Emily Stott, Carl D. Morrison, Shousong Cao, Arup Bhattacharya

**Affiliations:** ^1^Department of Cancer Biology, Roswell Park Cancer Institute, Buffalo, NY 14263, USA; ^2^Department of Immunology, Roswell Park Cancer Institute, Buffalo, NY 14263, USA; ^3^Department of Pathology, Roswell Park Cancer Institute, Buffalo, NY 14263, USA; ^4^Department of Cancer Prevention & Control, Roswell Park Cancer Institute, Buffalo, NY 14263, USA

## Abstract

Tumor differentiation enhances morphologic and microvascular heterogeneity fostering hypoxia that retards intratumoral drug delivery, distribution, and compromise therapeutic efficacy. In this study, the influence of tumor biologic heterogeneity on the interaction between cytotoxic chemotherapy and selenium was examined using a panel of human tumor xenografts representing cancers of the head and neck and lung along with tissue microarray analysis of human surgical samples. Tumor differentiation status, microvessel density, interstitial fluid pressure, vascular phenotype, and drug delivery were correlated with the degree of enhancement of chemotherapeutic efficacy by selenium. Marked potentiation of antitumor activity was observed in H69 tumors that exhibited a well-vascularized, poorly differentiated phenotype. In comparison, modulation of chemotherapeutic efficacy by antiangiogenic selenium was generally lower or absent in well-differentiated tumors with multiple avascular hypoxic, differentiated regions. Tumor histomorphologic heterogeneity was found prevalent in the clinical samples studied and represents a primary and critical physiological barrier to chemotherapy.

## 1. Introduction

Despite concerted efforts for more than five decades, curative response to chemotherapy remains elusive in majority of solid malignancies [1]. Efforts in discovering novel anticancer agents without similar efforts in understanding and overcoming physical barriers within tumors are unlikely to radically change chemotherapeutic efficacy in the clinic. Tumor being a heterogeneous three-dimensional composite of various components, factors at coarser physiological scales can and does influence tumor response [2]. For an optimum chemotherapeutic efficacy, the drug has to extravasate, diffuse to distant tumor cells, be transported inside the cells, bind to the specific target, and induce cell death. Only when an anticancer agent is able to affect each and every individual proliferating cancer cells in effectively inducing tumor cell death, can it result in a complete remission (CR) or a cure. Factors within the tumor microenvironment that impede drug delivery and distribution are likely to result in tumor regrowth and resistance irrespective of the drug's efficacy in vitro. 

Tumor drug delivery system constituted predominantly of abnormal vasculature, lack pericyte coverage, have abnormal branching patterns and shunt perfusion, prestasis, stasis and reversal of flow [3]. This dilated, chaotic and leaky vasculature with an intermittent or unstable blood flow contributes to an adverse and high intratumoral interstitial fluid pressure (IFP) that retards delivery and distribution of drugs from the vessels into the tumor [4]. We have previously demonstrated that differentiated regions in squamous cell carcinoma and adenocarcinoma do not contain blood vessel and contribute to heterogeneity in microvessel distribution that physiologically further retards an optimal intratumoral drug delivery and distribution [5–7]. Various strategies for improving drug penetration include improving tumor blood flow, enhancing vascular permeability, reducing IFP, and modifying the extracellular matrix [2]. For example, use of antiangiogenic agents has been shown to result in a favorable improvement in extent and quality of tumor perfusion while reducing vascular permeability and tumor IFP as a result of tumor vascular normalization [5, 7]. While there has been an ongoing effort in improving tumor drug penetration in order to improve therapeutic efficacy, newer approaches are required to overcome tumor architectural and morphologic barriers that foster therapeutic resistance by adversely affecting tumor drug delivery.

In our earlier studies, we have observed that high but nontoxic doses of antiangiogenic organoselenium compounds such as 5-methylselenocysteine (MSC) and selenomethionine (SLM) act as selective modulators of chemotherapeutic efficacy of a broad range of anticancer drugs (irinotecan, taxanes, platinum complexes, doxorubicin and cyclophosphamide) that are currently used in the clinic [8]. Administration of MSC (0.2 mg/mice/day per oral starting 7 days before irinotecan) in combination with irinotecan (100 mg/kg i.v. weekly × 4) was found to enhance therapeutic response from 20% and 30% CR with the drug alone to 100% CR in uniformly well-vascularized poorly differentiated colorectal carcinoma HCT-8 and head and neck squamous cell carcinoma (HNSCC) FaDu, respectively [8]. In contrast, this therapeutic synergy was less dramatic in well differentiated xenografts such as the colorectal adenocarcinoma HT-29 and HNSCC A253 (0% and 10% CR with the drug alone to 20% and 60% CR with the combination, resp.). In subsequent studies, we demonstrated that this therapeutic synergy was the result of enhanced tumor drug delivery and distribution as a consequence of an improved tumor vascular normalization and tumor IFP [5, 7]. 

In this study, we examined the influence of tumor histologic heterogeneity on the interaction between cytotoxic chemotherapy and selenium (Se) using a panel of surgical human tumor xenografts representing cancers of the head and neck (A253, well differentiated and poorly differentiated patient tumor derived HNSCC xenografts) and lung (H69, A549). Tumor differentiation status, microvessel density, interstitial fluid pressure, tumor blood, volume and permeability were correlated with the degree of enhancement of chemotherapeutic efficacy by selenium. Additionally, tissue microarray (TMA) analysis of human surgical samples was also performed to examine the occurrence and relevance of the observed biologic heterogeneity.

## 2. Materials and Methods

### 2.1. Tumor Model

The human cancer cell lines H69 (small cell lung cancer, SCLC), A549 (nonsmall cell epithelial lung carcinoma, NSCLC), and A253 (well differentiated head and neck squamous cell carcinoma) were originally obtained from American Type Culture Collection (Manassas, VA) and xenografts were established in ~8 week old female athymic nude mice (Foxn1^nu^, Harlan Sprague Dawley, Inc. Indianapolis, IN) as previously described [6]. Human poorly differentiated squamous cell carcinoma (SCC) surgical sample #17073 (maxillary sinus, PDSCC) and well differentiated SCC #16653 (larynx, WDSCC) were obtained in house at Roswell Park Cancer Institute and were maintained in SCID (C.B-Igh-1^b^IcrTac-Prkdc^scid^/Ros) mice. Mice were assessed for tumor growth using digital vernier calipers for measuring tumor burden (mm^3^) using the formulae: 1/2 (*L* × *W* ^2^), where *L* and *W* are the longest and shortest axis in mm as per established method [8]. All studies were performed in accordance with Institute Animal Care and Use Committee-approved protocols and each treatment group had a minimum of 4 animals per group.

### 2.2. Patient Samples of Cancer

Formalin/paraffin sections of human cancer surgical TMA containing 0.6 mm cores from head and neck, colorectal, and lung cancer were studied for presence or absence of differentiated structure, microvessel distribution (MVD), and tumor hypoxia. Tumor vessels were detected using CD34 marker for endothelial cells and hypoxia was determined using carbonic anhydrase IX (CAIX) staining as per methods described earlier [9].

### 2.3. Drugs and Chemicals

MSC or SLM (1 mg/mL, Sigma, St. Louis, MO) was administered orally as a sterile saline solution at the maximum tolerated dose of 0.2 mg/mouse/day [8] or 8 mg/kg/day for 14 days, starting at least 3 days after tumor implantation. Taxotere (Sanofi-Aventis, Bridgewater, NJ) was administered as a single intravenous dose of 60 mg/kg while irinotecan (Pharmacia & Upjohn, New York, NY) was administered at a weekly schedule of 100 or 200 mg/kg ×4. Doxorubicin (Bedford Laboratories, Bedford, OH) was administered as a single intravenous dose of 30 mg/kg, 24 hours after the MSC dose on day 14. Albumin-GdDTPA was obtained from the Contrast Media Laboratory, Department of Radiology (Dr. Robert C. Brasch), University of California at San Francisco (San Francisco, CA). For the combination chemotherapy with MSC/SLM, MSC or SLM was given 7 days before therapy and continued daily for 7 more days after the last dose of the chemotherapeutic drug.

### 2.4. Immunohistochemistry

MVD determination was done using CD31 in mouse and CD34 in human tumor TMAs. CD31 and alpha-smooth muscle actin (*α*-SMA) double staining was used to detect endothelial cells and pericytes, respectively, as described previously [5]. Briefly, 5–8 *μ*m cryosections were fixed in cold acetone (−20°C) for 15 minutes and following quenching with endogenous peroxidase were incubated with rabbit polyclonal SMA antibody (1 *μ*g/mL or 1/500) (Abcam, Cambridge, MA), biotinylated goat antirabbit secondary antibody (1/250) (Vector Labs) for 30 minutes, and CD31 antibody (B.D. Biosciences Pharmingen, Franklin Lakes, NJ) at a concentration of 10 *μ*g/mL for 60 minutes. An isotype-matched negative control was used in all cases. Immunostaining of tumor vasculature in the TMAs was done using antihuman CD34 (DAKO, Carpinteria, CA) used at 1/50 dilution for 90 minutes at room temperature followed by 30 minutes incubation with Streptavadin complex (Zymed Lab, Inc., San Francisco, CA) as per methods described earlier [5–7]; CAIX immunostaining, the primary antibody M75 (gift from Dr Pastorek, Institute of Virology, Slovak Republic) was used at 20 *μ*g/mL for 90 minutes followed by 30-minutes incubation with Streptavadin complex (Zymed Lab, Inc., San Francisco, CA) as described earlier [5–7]. Hypoxia inducible factor 1-*α* (HIF-1*α*) was detected with a multilayer, amplified method after antigen retrieval with Target Retrieval Solution (TRS, Dako Carpenteria, CA) in a pressure cooker. The method was developed and optimized at our Core Facility [10]. All immunohistochemical interpretation and analysis was carried out under the supervision of a board-certified and experienced pathologist (KT).

### 2.5. Magnetic Resonance Imaging

Tumor-bearing mice were imaged in a 4.7 T horizontal bore MR scanner (GE NMR Instruments, Fremont, CA). The imaging protocol and data analysis methods for estimating the vascular volume and permeability of tumors using the intravascular contrast agent, albumin-GdDTPA, have been previously described [11, 12]. The change in tumor T1-relaxation rate of tumors (ΔR1) was calculated over approximately 30–50 minutes post contrast. ΔR1 values were measured in the kidneys as a measure of vascular relaxation enhancement and used to compute vascular volume (*y*-intercept of the linear regression line) and permeability (slope of the linear regression line) tumor/blood fit. Image processing and analysis were carried out using commercially available medical imaging software Analyze PC, Version 7.0 (AnalyzeDirect, Lenexa, KS).

### 2.6. In Vivo Tumor IFP Measurements

MSC-induced changes in tumor IFP were monitored in A253, H69, and A549 xenografts in vivo real-time using microcatheter pressure transducers in externally accessible tumors and in normal tissue as per method described earlier [5, 7]. The interstitial fluid pressure within the tumor microenvironment was measured-using a modified “wick-in-needle” technique using custom-designed instrumentation broadly based upon an earlier published design [13]. Briefly, measurements were made with a MikroTip Catheter Transducer (Model SPR-524, Millar Instruments) via a 23.5 gauge wing-tipped infusion needle catheter. The transducer was interfaced to a PC using a pressure control unit (PCU-2000, Millar Instruments) via an USB analog-to-digital converter (Model DT9816 Data Translation, Marlboro MA). The software used to acquire the data was developed in the laboratory using DT Measure Foundry Ver. 4.0.7 (Data Translation, Marlboro, MA). The needle was inserted into the tumor, with measurements made every few millimeters; thus three to six measurements were made within each tumor, and the average value was used. Prior to each measurement, the apparatus was aspirated to ensure that no tissue was clogging the needle, and the instrument was zeroed. The instrument was calibrated before and after each experiment to ensure proper function, using a custom built water-column manometer. Mice with subcutaneous tumors (N = 4–8 per group) were used to assess the IFP measurements under anesthesia at time points equivalent to 24 hours post 14 days of MSC.

### 2.7. Determination of Intratumoral Doxorubicin Distribution Gradient

The influence of MSC on drug distribution gradient (*N* = 47 or more linear paths 90 pixels long using multiple sections from tumors) was assessed using fluorescence microscopy as described earlier [5]. Two hours post doxorubicin administration, animals were euthanized and the harvested tumor frozen and ~5–10 *μ*m thick frozen sections were used. An average of 4 maximum intensity projection images with a resolution of 0.23 *μ*m was acquired using identical acquisition parameters under 63× objective of Leica confocal microscope [5] and the digitized images were used further for image analysis.

### 2.8. Image Analysis

CD31 positive endothelial cell clusters in multiple high-power fields (400×) covering non-necrotic areas of the whole tumor sections were used to determine MVD. Tumor vascular maturation index (VMI) was derived by calculating the total number of CD31+ *α*-SMA+ areas and areas positive for CD31 alone in double-stained (CD31/*α*-SMA) tissue sections using Analyze (AnalyzeDirect, OverlandPark, KS) [5]. The mean intensity of doxorubicin autofluorescence at various distances away from the blood vessels was calculated using Analyze.

### 2.9. Statistical Analysis

Results are expressed as mean ± standard error of the mean and the differences between the mean of the groups were analyzed using unpaired two-tailed Student *t*-test (GraphPad Version 5.00, GraphPad Software, San Diego, CA). Linear regression analysis was carried out to determine statistical significance of DCE-MRI-measured parameters and intratumoral drug distribution gradient. A *P* value <.05 considered statistically significant.

## 3. Results

### 3.1. Histological Characteristics of Tumor Xenografts

**Figure 1:**
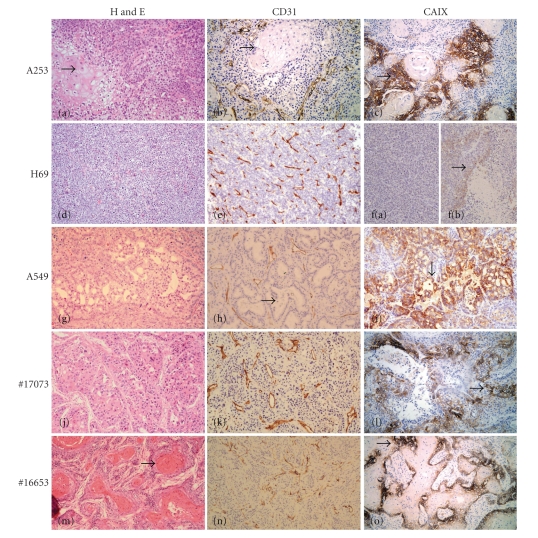
Photomicrographs of human cancer cell line xenografts A253, H69, A549 along with human HNSCC surgical samples-derived xenografts #17073 and #16653. (Left panels, H&E; middle panels, CD31 immunostaining to visualize microvessels; right panels, CAIX immunostaining to visualize tumor hypoxic regions; original magnifications, ×100). Poorly differentiated H69 (d) is uniformly well vascularised (e) and has no regions of hypoxia (f(a)) in the ~250 mg tumor but has perinecrotic hypoxic regions in larger (>2000 mg) tumor (f(b), *arrow*). Similar vascular arrangement (k) is seen in viable regions of the poorly differentiated HNSCC #17073 (PDSCC) which though has hypoxic regions ((l), *arrow*) around the many necrotic regions seen in the growing tumor. In contrast, A253 and A549 have differentiated regions ((a), *arrow* & (g)) that being avascular ((b), (h), *arrows)* are hypoxic ((c), (i), *arrows*). Surgical sample derived well differentiated HNSCC #16653(WDSCC) has a highly differentiated morphology due to presence of many well differentiated ((m), *arrow*) regions that are avascular (n) and hypoxic ((o), *arrow*).

While H69 (Figure 1(d)) is a poorly differentiated small cell lung carcinoma, HNSCC A253 (Figure 1(a)) and the non-small cell lung adenocarcinoma A549 (Figure 1(g)) are well and moderately differentiated tumors, respectively. CD31 immunostaining shows H69 (Figure 1(e)) to be uniformly well-vascularized while A253 (Figure 1(b)) and A549 (Figure 1(h)) xenografts have differentiated regions that are devoid of blood vessels and are thus consequently hypoxic as seen by presence of CAIX immunostaining (*arrows, *Figures 1(c) and 1(i), resp.). While very few cells were found to be CAIX positive in smaller H69 tumors (<1000 mg, Figure 1(f(a))), the larger H69 tumors (>2000 mg, Figure 1(f(b))) had the characteristic perinecrotic hypoxia. As shown in Figure 1(j), #17073 is a PDSCC while #16653 is a WDSCC (Figure 1(m)). The hallmark of surgical PDSCC xenografts was the presence of many necrotic regions throughout the tumor. These necrotic regions did not contain viable tumor cells or any vasculature and were surrounded by CAIX positive hypoxic cells (*arrow,* Figure 1(l)). Though the other regions in the PDSCC exhibited a uniform microvessel distribution (Figure 1(k)), these necrotic regions contribute to a histomorphological heterogeneity that is not conducive to an optimal intratumoral drug delivery and distribution. The surgical WDSCC xenograft contained several well differentiated regions (*arrow,* Figure 1(m)) that are without microvessels (Figure 1(n)) and are surrounded by rims of CAIX positive hypoxic proliferating cells (*arrow,* Figure 1(o)).

### 3.2. Effect of MSC on Tumor Growth

**Figure 2:**
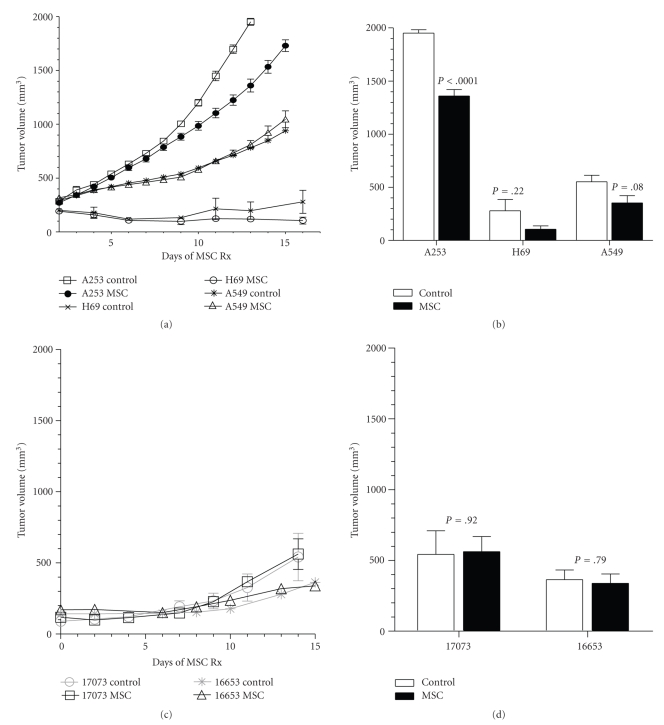
Effect of MSC on tumor growth ((a), (c)) and tumor volume ((b), (d)) at the end of 14 days treatment.

As shown in Figure 2(a), the HNSCC A253 grew at a faster pace than the lung xenografts H69 and A549. Treatment with MSC resulted in a reduction of tumor burden when compared to the untreated controls (Figure 2(b)) by 30%, 62%, and 36% in A253 (1952 ± 32.07 versus 1360 ± 59.75, *N* = 6), H69 (279.5 ± 106.60 versus 105 ± 31.84, *N* = 4) and A549 (552.50 ± 60.62 versus 353.40 ± 68.16, *N* = 4), respectively, albeit significantly only in A253 (*P* < .0001). Untreated PDSCC and WDSCC xenografts grew slower than control A253 tumors (Figure 2(c)). Treatment with MSC for 2 weeks did not lead to a significant reduction in tumor burden when compared to untreated controls in both PDSCC (541.9 ± 167.2 versus 561.80 ± 107.30, *N* = 6) and WDSCC (363.80 ± 68.53 versus 338.50 ± 65.63, *N* = 6–8) xenografts (Figure 2(d)).

### 3.3. Effect of MSC on MVD, IFP, and Drug Delivery

**Figure 3:**
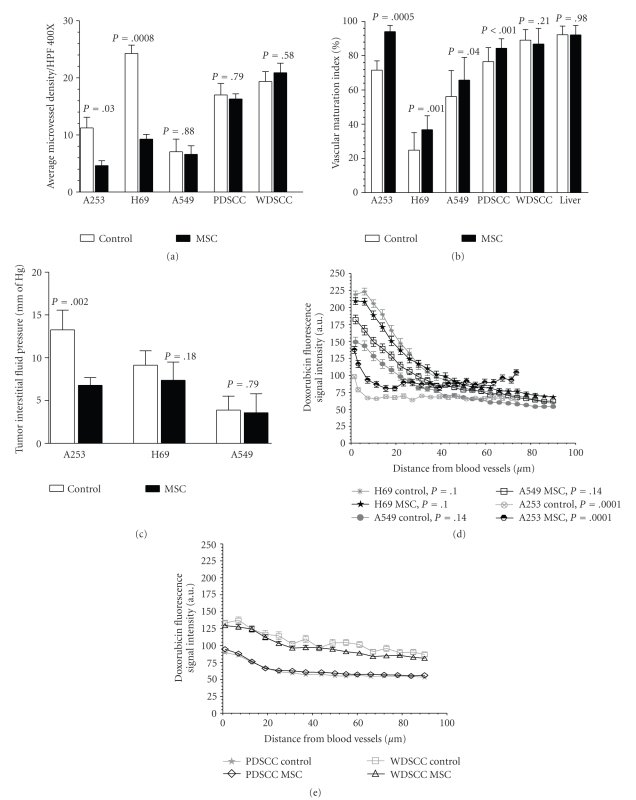
(a) Bar graphs show MVD counts per high-power field (HPF) (original magnification, 400×) in the untreated versus MSC-treated xenografts and surgical samples following 14 days of MSC treatment (0.2 mg/mouse/day). Significant reduction in MVD was seen in A253 and H69 xenografts after 14 days of MSC treatment with no changes seen in A549 xenografts or the surgical samples. (b) MSC led to an improved vascular maturation in A253, H69, A549 xenografts and PDSCC but did not induce any change in WDSCC and normal liver. (c) Treatment with MSC (0.2 mg/mouse/day × 14) led to a lowering of interstitial fluid pressure in A253 but not in H69 and A549 xenografts. (d) As a consequence, there was a significant improvement in tumor doxorubicin fluorescence intensity gradient in A253 xenografts but not in H69 or A549 xenografts. (e) MSC did not result in an improvement in tumor doxorubicin fluorescence intensity gradient in the surgical samples PDSCC and WDSCC.

Poorly differentiated untreated H69 xenografts were highly vascularized compared to the untreated well differentiated HNSCC A253 (*P* = .002) and NSCLC A549 (*P* = .008) tumors. As shown in Figure 3(a), the antiangiogenic effect of MSC (0.2 mg/mouse/day × 14) led to a 59%, 62%, and 6% reduction in MVD compared to untreated control tumors in A253 (4.63 ± 0.87 versus 11.23 ± 1.87, *P* = .03), H69 (9.28 ± 0.82 versus 24.29 ± 1.45, *P* = .008) and A549 (6.60 ± 1.50 versus 7.05 ± 2.23, *P* = .88) respectively. No differences in MVD of normal mouse liver tissue were observed (data not shown). Tumor vascular maturation index (VMI), that is, the percentage of endothelial cells associated with pericytes, showed an increase of 31.38%, 49.93% and 6.38% in A253, H69 and A549 xenografts, respectively (Figure 3(b)). Tumor IFP was higher in the untreated HNSCC xenograft A253 compared to the untreated lung tumor xenografts that were lower by 31% and 71% in H69 and A549, respectively. As shown in Figure 3(c), treatment with MSC led to a significant reduction in tumor IFP in A253 compared to the untreated controls (6.78 ± 0.45 versus 13.26 ± 1.15, *P* = .002) but this reduction was less dramatic in the lung xenografts—H69 (7.38 ± 0.75 versus 9.13 ± 0.84, *P* = .18) and A549 (3.58 ± 0.91 versus 3.89 ± 0.66, *P* = .79). The net effect of MSC on tumor vasculature and IFP was a significantly (*P* = .0001) increased intratumoral doxorubicin gradient in A253 xenografts while there was no significant increase in the overall drug gradient in the lung xenografts (Figure 3(d)). Interestingly, at a distance of ~90 *μ*m from tumor blood vessels, all the MSC-treated tumors showed a significantly higher doxorubicin concentration as measured by the fluorescence intensity compared to the non-MSC-treated A253 (91.38 ± 4.81 versus 66.90 ± 3.21, *P* = .001), H69 (68.06 ± 2.60 versus 59.17 ± 1.41, *P* = .003), and A549 (62.21 ± 1.84 versus 53.94 ± 1.58, *P* = .0008) xenografts. 

In contrast, the untreated human surgical samples of HNSCC had a higher MVD (17 ± 0.3 in PDSCC, and 19 ± 0.41 in WDSCC) compared to HNSCC A253 xenograft (11.23 ± 1.87) (Figure 3(a)). Though treatment with MSC did not lead to a significant reduction in MVD in PDSCC compared to untreated controls (16 ± 0.13 versus 17 ± 0.3), there was a significant increase in tumor vascular maturation (84.39 ± 1.06 versus 76.57 ± 1.48, *P* < .001) (Figure 3(b)). In WDSCC, MSC did not lead to any reduction in MVD (20.90 ± 0.35 versus 19.36 ± 0.4) or improvement in VMI (86.82 ± 1.45 versus 89.10 ± 1.03). The intratumoral doxorubicin gradient was not significantly different in both the surgical samples (Figure 3(e)). At a distance of ~90 *μ*m from tumor blood vessels, MSC-treated tumors showed a significantly higher doxorubicin concentration as measured by the fluorescence intensity compared to the non-MSC treated PDSCC (56.13 ± 0.94 versus 52.96 ± 0.94, *P* = .047). A similar increase was not seen in the well differentiated WDSCC at ~90 *μ*m from tumor blood vessels. Instead, a significant increase in doxorubicin fluorescence intensity was seen at a distance of ~50 *μ*m within the avascular well differentiated hypoxic foci in the MSC-treated tumors compared to the non-MSC-treated tumors (63.62 ± 2.11 versus 56.32 ± 1.96, *P* = .02).

### 3.4. Influence of Histologic and Vascular Heterogeneity on Tumor Vascular Response to Selenium

**Figure 4:**
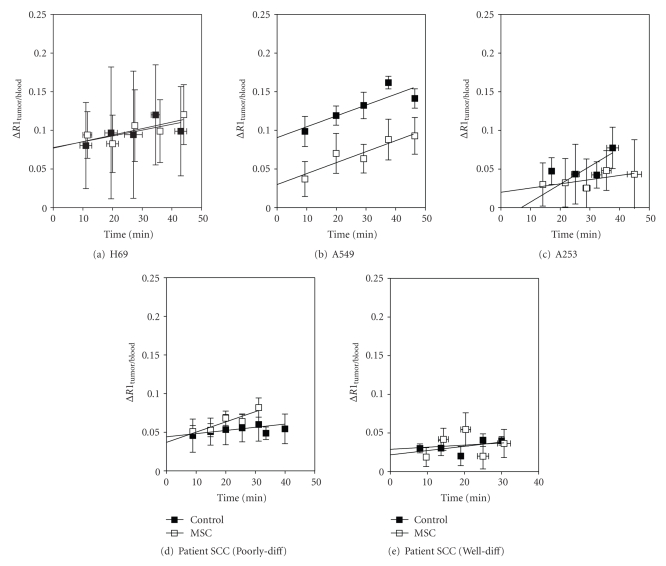
Change in relative vascular permeability and vascular volume as a result of treatment with MSC in H69 (a), A549 (b), A253 (c), PDSCC (d), and WDSCC (e) tumors as assessed by DCE-MRI.

In order to examine changes in vascular function following selenium treatment, noninvasive MRI was utilized. Changes in vascular volume and permeability were estimated in tumor-bearing mice following treatment with MSC (0.2 mg/day × 14) and compared to untreated controls. As shown in Figure 4, only A549 tumors showed a significant reduction (*P* < .001) in vascular volume compared to untreated control tumors (*n* = 4 per group). No significant differences in tumor vascular volume and permeability were observed between control and selenium-treated mice for H69 (*P* > .05; *N* = 3 controls, *N* = 4 MSC), A253 (*P* > .05; *N* = 9 controls, *N* = 7 MSC), poorly differentiated (*P* > .05; *N* = 8 controls, *N* = 12 MSC), and the well differentiated patient tumor-derived HNSCC xenografts (*P* > .05; *N* = 6 per group).

### 3.5. Modulation of Antitumor Activity

**Figure 5:**
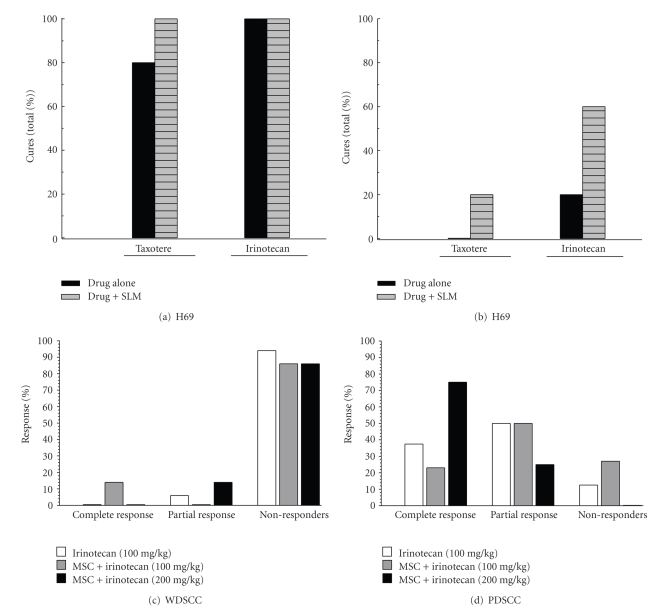
Response to cytotoxic drugs alone and in combination with MSC in H69 lung xenografts when treatment was started at ~100 mm^3^ tumor volume (a), at ~1000 mm^3^ tumor volume (b), and in human surgical samples of HNSCC WDSCC and PDSCC.

We had earlier reported that MSC (0.2 mg/mouse/day p.o. starting 7 days prior to and continuing daily till end of chemotherapy) in combination with the maximum tolerated dose of irinotecan (100 mg/kg i.v. weekly × 4) resulted in an increase in cure rates (CR) in A253 xenografts from 10% with irinotecan (100 mg/kg weekly × 4 i.v.) alone to 60% with the combination [8]. As can be seen in Figure 5(a), the small cell lung cancer xenograft H69 gives a 100% CR either with irinotecan (100 mg/kg weekly × 4 i.v.) alone or in combination with SLM. The CR with taxotere (60 mg/kg × 1 i.v) alone was 80% that increased to 100% when used in combination with SLM. These high cure rates were obtained when treatments were initiated at ~100 mm^3^ size of tumor in H69 tumor xenografts. H69, a uniformly well vascularised tumor with relatively small pockets of necrosis, is an attractive preclinical model for determining the influence of tumor morphological heterogeneity on synergistic chemotherapeutic efficacy in combination with antiangiogenic agents. To determine if this activity of combination treatment was persistent against larger tumors, studies were carried out using larger H69 tumors (~1000–1500 mm^3^). These large H69 tumors contain multiple necrotic regions surrounded by hypoxic CAIX positive tumor cells (Figure 1(f(b)) and, thus, are less homogeneous compared to the smaller (<1000 mg) H69 tumors. As seen in Figure 5(b), a CR of 0% and 20% with taxotere alone and in combination with SLM and a CR of 20% and 60% with irinotecan alone and in combination with SLM, respectively were achieved. In contrast, the moderately differentiated non-small cell lung carcinoma A549 had 0% cures either with the drug alone or in combination with MSC.

In order to further investigate this therapeutic efficacy of Se in combination chemotherapy, 2-patient tumor-derived HNSCC xenografts with varying differentiation status were used. Both xenografts were strongly positive for hypoxia as determined by CAIX immunostaining. As can be seen in Figure 5(c) and Figure 5(d), treatment with irinotecan (100 mg/kg weekly × 4 i.v.) alone gave a cure rate of 0% and 37.5% in WDSCC and PDSCC, respectively. In combination with MSC there was no significant improvement in CR except in WDSCC where the CR increased to 14%. Since our earlier studies [8] have indicated the possibility of dose escalation to double the normal MTD of irinotecan, due to the additional chemoprotective properties of MSC, we used this higher dose of irinotecan (200 mg/kg weekly × 4, i.v.) in another group of mice bearing these human cancer surgical samples. As can be seen in Figure 5(d), MSC in combination with this higher irinotecan dose resulted in a higher response of 75% CR and 25% partial response in PDSCC. No such improvement in response was seen in the surgical WDSCC. Thus, enhancement in therapeutic efficacy by antiangiogenic MSC in combination of chemotherapy is higher in tumors that are relatively more homogeneous versus in tumors that are highly heterogeneous such as in the WDSCC.

### 3.6. Patient Samples of HNSCC, Colorectal, and Lung Cancer

**Figure 6:**
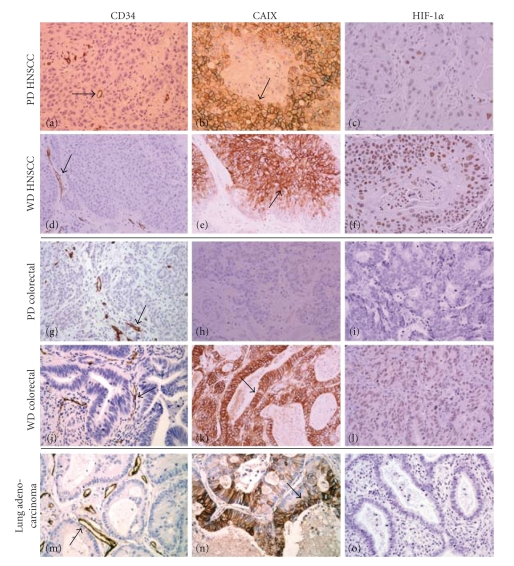
Photomicrographs of tumor tissue microarrays (TMAs) from surgical samples of head and neck squamous cell, colorectal, and lung carcinoma. (Left panels, CD34 immunostaining to visualize microvessels; middle panels, CAIX immunostaining and right panels, HIF-1*α* immunostaining (brown, nuclear) to visualize tumor hypoxic regions; original magnifications, ×200). Poorly differentiated TMAs are relatively uniformly well vascularised ((a), (g), *arrows*) with some regions of hypoxia as measured by CAIX ((b), *arrow*, (h)) and HIF-1*α* ((c), (i)). In contrast, the well differentiated tumors have large differentiated regions that are avascular ((d), (j), (m), *arrows*) and more strongly hypoxic as seen with CAIX ((e), (k), (n), *arrows*) and HIF-1*α* ((f), (l)) immunostaining. Most lung adenocarcinomas were negative for HIF-1*α* staining (o).

In order to determine the relevance of tumor morphologic heterogeneity in influencing efficacy of antiangiogenic therapy such as MSC/SLM, we studied the prevalence of architectural heterogeneity in solid tumor malignancies in terms of tumor cell differentiation, vascularization, and hypoxia using TMAs derived from patient surgical cancer specimens from three different disease sites—head and neck, colorectal, and lung. As summarized in Table 1, 51% of HNSCCs are poorly differentiated while 41% of HNSCCs are well differentiated (41%). Most (47%) HNSCC show presence of hypoxia marker CAIX. Figures 6(a) and 6(d) shows the representative tumor vascular distribution (*arrow, brown*) in poorly and well differentiated HNSCC with regions of hypoxia stained for CAIX immunostaining (Figures 6(b) and 6(e)) and HIF-1*α* (Figures 6(c) and 6(f)). In colorectal cancers, the majority were found to be moderately differentiated cancers (79%) and hypoxic (CAIX, 63%) (Table 1). Figures 6(g) and 6(j) show typical distribution of tumor MVD (*arrow, brown*) with regions of hypoxia stained for CAIX (Figure 6(k), *arrow, brown*) and HIF-1*α* (Figure 6(l), *brown*) immunostaining in the well differentiated colorectal cancers. NSCLC also presented itself predominantly (86%) as a moderately differentiated cancer with 41% of tumors being hypoxic (CAIX positive). Figure 6(m) shows the distribution of vessels (*brown*) in lung cancer (*arrow, brown*) while Figure 6(n) and 6(o) depict the distribution of hypoxia as assessed using CAIX (*brown, arrow*) and HIF-1*α*, respectively in lung cancers. The majority of solid malignancies studied was found to have the hallmark of a heterogeneous tumor architecture that is not conducive for an optimal intratumoral drug delivery and distribution and, thus, will not respond optimally to monotherapy or to the synergistic modulation by an antiangiogenic agents such as MSC/SLM used in combination setting.

## 4. Discussion

The effectiveness of traditional and novel anticancer agents is limited by physical barriers that compromise tumor drug delivery at therapeutically meaningful concentrations. Our knowledge and understanding of tumor vasculature and hypoxia has increased over the past few decades, but if this understanding is not tied to the morphologic or cellular heterogeneity arising out of tumor tissue differentiation, progress in treating solid malignancies will remain inadequate. Drug penetration in the tumor tissues is by convection and/or diffusion. Convection depends on both hydrostatic and osmotic pressure gradients between vascular space and interstitial space, vascular permeability, surface area; and the volume and structure of extracellular matrix [2]. Diffusion is determined by concentration gradients. Since tumor IFP is often elevated to the level of microvascular pressure, it retards extravasation of macromolecules that must penetrate distances of up to 200 *μ*m within the tumors in order to reach all viable cells [2]. While many studies have reported an association between low IFP and therapeutic response [14, 15], some others have not found such association [2, 16]. Obviously, a lowered IFP by itself may not be the panacea for improving drug delivery and therapeutic response. 

Differentiated cells do not lead to tumor propagation and metastasis but can result in a morphologic heterogeneity that hinders optimal intratumoral drug delivery [6]. While many studies have focused on the individual cancer cells, stromal cells and the vascular network, there are few studies on the effect of tumor histomorphologic heterogeneity in drug resistance. Tumor tissue architecture is an important factor that influences drug delivery. For instance, the higher cell density in differentiated regions of tumors prevents the sprouting and growth of blood vessels [6]. This combined with a reduced interstitial space and volume of extracellular matrix results in lower intratumoral drug penetration [2].

SCC and adenocarcinoma are the most common histological types of solid malignancies. In well differentiated forms of SCC, tumor cells nests are often observed with a closely packed arrangement of individual tumor cells without stroma or microvessels. Tumor cells in these differentiated regions (Figure 1(a), *arrow*) or the lumen structure (Figure 1(h), *arrows*) seen in adenocarcinoma do not receive adequate blood supply causing hypoxia, limited drug delivery and resulting in sanctuaries for proliferating cells that escape therapy. In contrast, the poorly differentiated parts of these tumors or in tumors that are wholly poorly differentiated, microvessels are uniformly distributed providing blood supply to most such parts of the tumors. In our study, MSC treatment led to a lower tumor burden in all three human tumor xenografts albeit only significantly in A253 xenografts. This effect was not seen in the human surgical samples of head and neck cancers-PDSCC and WDSCC. Greater optimal therapeutic synergy of antiangiogenic Se compounds in combination to anticancer drug was observed in the morphologically less heterogeneous large human SCLC cell line xenograft H69 compared to more heterogeneous NSLC A549 that had a lower, relatively normalized MVD, IFP, or an improved drug delivery. In contrast, A253 despite being well differentiated shows a better response to the combination therapy as reported earlier [8]. This is as a consequence of MSC-induced vascular modulation that led to a significant lowering of MVD, an improved VMI and IFP, thereby resulting in an increased intratumoral drug gradient. The heterogeneous differentiated human NSCLC A549 with a lower but relatively matured MVD did not show a marked modulation to antiangiogenic Se in terms of reduction in MVD, IFP or an improved VMI. Hence, it did not respond with a higher efficacy to the combination chemotherapy with Se. Interestingly, in SCLC H69, the synergy persisted even when the tumors were allowed to grow to ~1–1.5 g before the start of therapy. H69, a representative SCLC with uniformly poorly differentiated, well-vascularised tumor, contains few small necrotic foci and had the least morphologic heterogeneity in terms of its cellular architecture and vascular distribution amongst the xenografts studied. As shown in Figure 1(d), the more homogeneous morphologic feature of this tumor facilitates a better drug penetration and distribution within the tumor. This translates into a better therapeutic response. We had earlier reported similar results in the poorly differentiated human tumor xenografts HNSCC FaDu and colorectal HCT-8 that has a similar uniform vasculalarized homogeneous morphology and in which treatment with MSC resulted in a significant modulation of the vascular parameters and IFP [5, 7]. The antiangiogenic effect in terms of reduction in MVD was the highest in H69 (62%) and A253 (59%) and was minimal in A549. Despite a significant reduction in MVD and a dramatic improvement in VMI (49.93%), there was no significant difference in IFP or in the vascular parameters assessed with DCE-MRI in H69 xenografts. In all three tumor xenografts, treatment with MSC led to increased doxorubicin fluorescent intensity at a distance of ~90 *μ*m from vessel wall. These MSC-induced changes in vascular parameters and IFP resulted in a significant increase in response rates in H69 and A253 xenografts. Despite the minimal reduction in MVD, MRI revealed a statistically significant reduction in vascular volume of A459 tumors following selenium treatment (Figure 4). It is plausible that this dramatic reduction in perfusion contributes to decreased chemotherapeutic delivery leading to a minimal therapeutic response with combination therapy in this tumor model. 

SCLC, being poorly differentiated, homogeneous, and well vascularized, is sensitive to chemotherapy. Regrowth and resistance in the clinic has been associated with cellular differentiation in these cancers induced in part by the agents themselves [17]. Thus, an effective first line chemotherapeutic regime that can kill most of the cancer cells is likely to be more efficacious in such tumors and the use of antiangiogenic agents such as Se in the combinatorial setting is likely to be the most promising especially in chemo-naive patients. In contrast, the heterogeneous surgical samples of HNSCC did not show any significant MVD reduction by Se. The improvement in VMI was significant but modest (~8%) in the poorly differentiated surgical sample #17073 but was not significant in well differentiated #16653. Both these surgical samples did not show any MSC-induced increase in doxorubicin concentration gradient immediately adjacent to tumor vasculature and MSC did not result in an enhanced synergistic therapeutic response of irinotecan at its MTD in both these tumors. Unlike the poorly differentiated and uniformly vascularized FaDu, HCT8, and H69 xenografts, PDSCC is relatively less homogeneous due to the presence of irregular and large regions of hypoxia and necrosis that does not facilitate therapeutic exposure of drugs in meaningful concentration to all proliferating cancer cells (Figure 1(l)). Since Se has chemoprotective effect [8] and allows for dose escalation to higher than MTD, we treated a subset of the mice bearing these surgical specimens with MSC in combination with double the MTD of irinotecan. At this dose, MSC did result in a significant increase in CR (75%) in the surgical sample of PDSCC but showed no change in the surgical sample WDSCC. The histological structure of WDSCC is extremely heterogeneous due to the presence of many keratinized well differentiated avascular (Figure 1(m), *arrow*), hypoxic regions (Figure 1(o), *arrow*). Such regions do not allow penetration of an effective drug in a therapeutically meaningful concentration in the rim region surrounding these differentiated regions. This causes proliferating cells within these rims to escape therapy and allow tumor repopulation and regrowth [6]. Further, the remnant tumor after therapy generally contained mainly differentiated regions remaining as survivor—a pointer to their contribution to drug resistance [6].

As previously reported [18], poorly differentiated tumor xenografts such as FaDu and HCT-8 have a slightly faster doubling time (~2.5 to 3 days) compared to their well differentiated counterparts such as A253 and HT-29 (~3.5 days), respectively. In our studies, the PDSCC had a similar doubling time of 3.5–4 days while the WDSCC had a doubling time of more than 5 days (Figure 2(c)). The lung cancer xenografts had a slower proliferation rate with the poorly differentiated SCLC xenografts H69 showing a doubling time of 6.1 days [19] while the differentiated NSCLC was found to have a doubling time of ~8.3 days in our studies (Figure 2(a)). It is likely that due to the presence of more uniformly distributed and often higher number of blood vessel, the poorly differentiated tumors tend to proliferate and grow faster. Proliferating cells are more sensitive to chemotherapy while the uniform distribution of vessels in poorly differentiated tumors makes the tumor easily accessible to anticancer drugs especially after tumor vascular normalization by antiangiogenic agents including MSC/SLM. From the results, one can surmise that chemomodulation of an antiangiogenic agent such as MSC will be most optimal in tumors where it can significantly reduce MVD, increase VMI, and as a consequence lead to a reduced IFP. In tumors such as the A549 and WDSCC where the untreated controls showed a presence of either high (WDSCC) or a low (A549) MVD, and the addition of the antiangiogenic agent did not change the VMI, the chemomodulation effect of antiangiogenic agents in combination chemotherapy is unlikely to lead to a significantly high response. The higher the degree of tumor morphologic heterogeneity, the less likely is the synergistic effect of antiangiogenic agents in combination chemotherapy. There remains an urgent need in developing novel agents that can abrogate tumor morphological heterogeneity in order to increase chemotherapeutic response in the clinic.

Similar to observations by others, our results indicate that CAIX and HIF-1*α* do not always co-localize in the same tumor regions [20, 21]. CAIX is a more robust indicator of physiologic hypoxia while HIF-1*α* can often be transiently upregulated by acute hypoxia or by other non-hypoxia related factors [22].

The results of our TMA analysis, even with limited tissue, indicate presence of histological architectural heterogeneity in majority of HNSCC, colorectal and lung cancers. This heterogeneity is not conducive for optimal intratumoral drug delivery and distribution leading to the suboptimal chemotherapeutic response seen in the clinic. It is also likely to compromise synergistic efficacy of antiangiogenic agents in combination with anticancer agents. Morphologically homogeneous tumors such as SCLC, FaDu, and HCT-8 are more likely to respond to antiangiogenic agents in combinatorial chemotherapy setting especially in chemo-naive cases. 

Currently available antiangiogenic agents are limited by host tissue toxicity, are cost prohibitive [23], and suffer from the lacunae that most tumors can easily overcome the blockade of a single specific proangiogenic molecule through bypassing onto other proangiogenic markers/pathways. In fact, recent reports suggest an alarming trend towards increased metastatic and aggressive disease in the tumors surviving antiangiogenic therapy in both the clinical and preclinical model [24, 25]. Since Se is part of the mammalian physiology, it is relatively well tolerated and affects multiple upstream targets such as HIF-1*α*, Cox-2, and iNos [26] important for cancer survival and progression and is likely to have a better success as an antiangiogenic agent in combination chemotherapy [27]. Inhibition of HIF-1*α*, a critical master gene that regulates tumor angiogenesis, growth, survival, and resistance, has been shown recently to be through downregulation of reactive oxygen species and stabilization of prolylhydoxylase 2 and 3 by MSC [28]. This inhibition results in the observed antiangiogenic effects of MSC besides sensitizing the small but therapy resistant population of HIF-1*α* positive, hypoxic cell population to the cytotoxic effects of the chemotherapeutic agents used in the combination therapy with MSC [28]. Unlike other antiangiogenic agents, Se also asserts its anticancer efficacy independent of antiangiogenic effects through various mechanisms such as redox cycling, altering protein-thiol redox status and methionine mimicry [29]. Since the dose levels of Se used in the preclinical studies have been attained in the clinic [30], it is a clinically viable antiangiogenic agent that can enhance therapeutic efficacy of chemotherapy in various solid malignancies especially those with a relatively well-vascularised and homogenous tumor architecture. Further, the protective effect of Se on healthy tissues from the adverse effects of cytotoxic drugs [8] allows for drug dose escalation to higher than their MTDs. Our current data and our earlier published data [5, 7] demonstrate that a nontoxic dose of Se is a promising antiangiogenic agent for use in combination chemotherapy especially against solid tumors with little or no morphological heterogeneity such as what is seen in SCLC H69, HNSCC FaDu and the colorectal cancer HCT-8.

## Figures and Tables

**Table 1:** Prevalence of histomorphologic heterogeneity in human cancers.

Cancer disease site	Total evaluable tumors	Tumor differentiation status			CAlX immunostaining			HIF-1*α* immunostaining		
		(% of Total)			(% of Total)			(% of Total)		
		Well Diff	Mod. Diff	Poorly Diff	<33%	33–67%	>67%	<33%	33–67%	>67%
Head and neck	198	41% (72% CAIX+)	8% (50% CAIX+)	51% (38% CAIX+)	25%	8%	14%	9%	4%	8%
					Total—47% of all tumors			Total—21% of all tumors		

Colorectal	57	12% (57% CAIX+)	79% (64% CAIX+)	9% (60% CAIX+)	28%	5%	30%	7%	2%	12%
					Total—63% of all tumors			Total—21% of all tumors		

Lung	102	3% (0% CAIX+)	86% (42% CAIX+)	11% (36% CAIX+)	23%	10%	8%	3%	1%	0%
					Total—41% of all tumors			Total—4% of all tumors		

## References

[1] Savage P, Stebbing J, Bower M, Crook T (2009). Why does cytotoxic chemotherapy cure only some cancers?. *Nature Clinical Practice Oncology*.

[2] Tredan O, Galmarini CM, Patel K, Tannock IF (2007). Drug resistance and the solid tumor microenvironment. *Journal of the National Cancer Institute*.

[3] Carmeliet P, Jain RK (2000). Angiogenesis in cancer and other diseases. *Nature*.

[4] Jain RK (2001). Normalizing tumor vasculature with anti-angiogenic therapy: a new paradigm for combination therapy. *Nature Medicine*.

[5] Bhattacharya A, Seshadri M, Oven SD, Tóth K, Vaughan MM, Rustum YM (2008). Tumor vascular maturation and improved drug delivery induced by methylselenocysteine leads to therapeutic synergy with anticancer drugs. *Clinical Cancer Research*.

[6] Bhattacharya A, Tóth K, Mazurchuk R (2004). Lack of microvessels in well-differentiated regions of human head and neck squamous cell carcinoma A253 associated with functional magnetic resonance imaging detectable hypoxia, limited drug delivery, and resistance to irinotecan therapy. *Clinical Cancer Research*.

[7] Bhattacharya A, Tóth K, Sen A (2009). Inhibition of colon cancer growth by methylselenocysteine-induced angiogenic chemomodulation is influenced by histologic characteristics of the tumor. *Clinical Colorectal Cancer*.

[8] Cao S, Durrani FA, Rustum YM (2004). Selective modulation of the therapeutic efficacy of anticancer drugs by selenium containing compounds against human tumor xenografts. *Clinical Cancer Research*.

[9] Bhattacharya A, Tóth K, Durrani FA (2008). Hypoxia-specific drug tirapazamine does not abrogate hypoxic tumor cells in combination therapy with irinotecan and methylselenocysteine in well-differentiated human head and neck squamous cell carcinoma A253 xenografts. *Neoplasia*.

[10] Vaughan MM, Tóth K, Chintala S, Rustum YM (2010). Double immunohistochemical staining method for HIF-1a and its regulators PHD2 and PHD3 in formalin fixed paraffin embedded tissues. *Applied Immunohistochemistry and Molecular Morphology*.

[11] Demsar F, Roberts TPL, Schwickert HC (1997). A MRI spatial mapping technique for microvascular permeability and tissue blood volume based on macromolecular contrast agent distribution. *Magnetic Resonance in Medicine*.

[12] Seshadri M, Mazurchuk R, Spernyak JA, Bhattacharya A, Rustum YM, Bellnier DA (2006). Activity of the vascular-disrupting agent 5,6-dimethylxanthenone-4-acetic acid against human head and neck carcinoma xenografts. *Neoplasia*.

[13] Ozerdem U, Hargens AR (2005). A simple method for measuring interstitial fluid pressure in cancer tissues. *Microvascular Research*.

[14] Heldin C-H, Rubin K, Pietras K, Ostman A (2004). High interstitial fluid pressure—an obstacle in cancer therapy. *Nature Reviews Cancer*.

[15] Curti BD, Urba WJ, Alvord WG (1993). Interstitial pressure of subcutaneous nodules in melanoma and lymphoma patients: changes during treatment. *Cancer Research*.

[16] Herrera FG, Chan P, Doll C (2007). A prospective phase I-II trial of the cyclooxygenase-2 inhibitor celecoxib in patients with carcinoma of the cervix with biomarker assessment of the tumor microenvironment. *International Journal of Radiation Oncology Biology Physics*.

[17] Brambilla E, Moro D, Gazzeri S (1991). Cytotoxic chemotherapy induces cell differentiation in small-cell lung carcinoma. *Journal of Clinical Oncology*.

[18] Azrak RG, Cao S, Slocum HK (2004). Therapeutic synergy between irinotecan and 5-fluorouracil against human tumor xenografts. *Clinical Cancer Research*.

[19] Kanashiro CA, Schally AV, Zarandi M, Hammann BD, Varga JL (2004). Suppression of growth of H-69 small cell lung carcinoma by antagonists of growth hormone releasing hormone and bombesin is associated with an inhibition of protein kinase C signaling. *International Journal of Cancer*.

[20] Mayer A, Höckel M, Vaupel P (2005). Carbonic anhydrase IX expression end tumor oxygenation status do not correlate at the microregional level in locally advanced cancers of the uterine cervix. *Clinical Cancer Research*.

[21] Hedley D, Pintilie M, Woo J (2003). Carbonic anhydrase IX expression, hypoxia, and prognosis in patients with uterine cervical carcinomas. *Clinical Cancer Research*.

[22] Kaluz S, Kaluzová M, Liao S-Y, Lerman M, Stanbridge EJ (2009). Transcriptional control of the tumor- and hypoxia-marker carbonic anhydrase 9: a one transcription factor (HIF-1) show?. *Biochimica et Biophysica Acta*.

[23] Verheul HMW, Pinedo HM (2007). Possible molecular mechanisms involved in the toxicity of angiogenesis inhibition. *Nature Reviews Cancer*.

[24] Ellis LM, Reardon DA (2009). Cancer: the nuances of therapy. *Nature*.

[25] Paez-Ribes M, Allen E, Hudock J (2009). Antiangiogenic therapy elicits malignant progression of tumors to increased local invasion and distant metastasis. *Cancer Cell*.

[26] Yin M-B, Li Z-R, Tóth K (2006). Potentiation of irinotecan sensitivity by Se-methylselenocysteine in an in vivo tumor model is associated with downregulation of cyclooxygenase-2, inducible nitric oxide synthase, and hypoxia-inducible factor 1*α* expression, resulting in reduced angiogenesis. *Oncogene*.

[27] Whanger PD (2004). Selenium and its relationship to cancer: an update. *The British Journal of Nutrition*.

[28] Chintala S, Tth K, Cao S (2010). Se-methylselenocysteine sensitizes hypoxic tumor cells to irinotecan by targeting hypoxia-inducible factor 1*α*. *Cancer Chemotherapy and Pharmacology*.

[29] Jackson MI, Combs GF (2008). Selenium and anticarcinogenesis: underlying mechanisms. *Current Opinion in Clinical Nutrition and Metabolic Care*.

[30] Fakih MG, Pendyala L, Brady W (2008). A phase I and pharmacokinetic study of selenomethionine in combination with a fixed dose of irinotecan in solid tumors. *Cancer Chemotherapy and Pharmacology*.

